# Revisiting Inductively Coupled Wireless Coils in MRI: Mitigating Over‐Coupling With Preamplifiers

**DOI:** 10.1002/mrm.70485

**Published:** 2026-06-21

**Authors:** Ming Lu, John C. Gore, Xinqiang Yan

**Affiliations:** ^1^ Vanderbilt University Institute of Imaging Science, Vanderbilt University Medical Center Nashville Tennessee USA; ^2^ Department of Biomedical Engineering Vanderbilt University Nashville Tennessee USA; ^3^ Department of Radiology and Radiological Sciences Vanderbilt University Medical Center Nashville Tennessee USA; ^4^ Department of Electrical and Computer Engineering Vanderbilt University Nashville Tennessee USA

**Keywords:** impedance mismatch, inductively coupled coils, preamplifier decoupling, RF coils, wireless coils

## Abstract

**Purpose:**

Inductively coupled coils enhance local MRI sensitivity, yet strong coupling with nearby primary coils typically causes resonance splitting and impedance mismatch, which are traditionally considered detrimental. This work investigates why inductively coupled coils can still function effectively even in the presence of severe coupling and clarifies the role of modern receive preamplifiers in mitigating coupling effects.

**Methods:**

Bench experiments were performed using primary coils (10 and 15 cm) and secondary inductively coupled coils (3–9 cm) tuned to the same Larmor frequency at 1.5, 3, and 7 T. Resonance characteristics and primary‐coil impedance variations were evaluated under open‐circuit, 50‐Ω, and low‐input‐impedance preamplifier terminations. MRI validation was conducted at 7 T without retuning the primary coil after introducing a closely positioned, inductively coupled coil, while intentionally varying preamplifier decoupling conditions.

**Results:**

Open‐circuit and 50‐Ω terminations produced pronounced resonance splitting and significant impedance distortion. In contrast, low‐input‐impedance preamplifier termination preserved the inductively coupled coil resonance despite strong coupling. Although the primary‐coil impedance shifted substantially, it remained within acceptable noise‐figure contours, resulting in negligible SNR penalty. Degraded preamplifier decoupling led to a 21%–23% SNR reduction.

**Conclusion:**

Modern preamplifiers fundamentally alter coupled‐coil behavior, enabling inductively coupled coils to operate near primary coils without significant SNR degradation and simplifying inductively coupled coil design.

## 1 Introduction

Inductively coupled coils, also termed passive resonators or wireless coils, have seen a resurgence in interest due to their structural simplicity and design flexibility. In this study, the secondary (inductively coupled) coil refers to the signal reception coil that works in conjunction with the primary coil through inductive coupling. These inductively coupled coils can significantly enhance the RF field by enabling close proximity to the tissue and/or allowing for optimized resonator sizes and current patterns.

The design of inductively coupled coils dates back to the early days of MRI [1, 2]. A typical application involves using a small inductively coupled coil as an enhancer for a large primary coil directly connected to the scanner. The primary coil is usually the built‐in body coil [3, 4, 5, 6, 7, 8, 9, 10, 11, 12, 13], a large‐volume coil [14, 15, 16], or a traveling‐wave antenna at ultrahigh field [17, 18]. Because the primary coil is typically much larger and far from the inductively coupled coil, the risk of over‐coupling is minimized, allowing the inductively coupled coil to be treated largely independently. Here, “over‐coupling” refers to the regime in which the mutual coupling between the coils is large enough that the coupled system supports two clearly split resonant eigenmodes rather than a single resonance at the Larmor frequency (Figure S1).

This situation changes, however, when the primary coil is a local receive coil. In this configuration, the comparable size and close proximity of the two coils result in strong coupling, and the system must be analyzed as a unified whole. As analyzed by Wright et al. and Hoult et al., the impedance of the primary coil should be adjusted to account for the presence of the inductively coupled coil, and the inductively coupled coil is often tuned slightly off the Larmor frequency [19, 20]. These adjustments are dependent on the mutual coupling strength, and thereby the relative positioning of the inductively coupled coil and primary coil, which may limit the flexibility of repositioning the inductively coupled coil.

Recently, Alipour et al. and Okada et al. demonstrated sensitivity improvements with frequency‐offset or structurally distinct coils [21, 22]. More recently, Zhu et al. and Lu et al. showed that same‐frequency, same‐structure inductively coupled coils can provide significant imaging benefits [23, 24, 25]. Subsequently, Lu et al. integrated ∼5‐cm‐diameter inductively coupled coils with a tight‐fitting 32‐channel head array at 7 T [26]. Despite the few‐centimeter separation from the ∼10‐cm‐diameter primary elements, which typically causes severe over‐coupling, significant local SNR gains were achieved [26]. Several other groups have reported similar findings regarding SNR enhancement using inductively coupled coils paired with either local primary coils or volume coils in different MRI applications [27, 28, 29, 30, 31, 32, 33].

These practical findings suggest that over‐coupling may not be a prohibitive concern for deployment. Two contributing factors are: the well‐established preamplifier decoupling mechanism since Roemer et al. [34], and the large tolerance of modern preamplifiers to variations in the coil's input impedance. First, their low input impedance preserves the resonant behavior of the inductively coupled coils, even when they are closely positioned to the primary coil. Second, their robust noise performance ensures a consistently low noise figure and substantial gain, even when the coil impedance deviates significantly from the nominal 50 Ω match.

## 2 Methods

### 2.1 Review of Over‐Coupling When an Inductively Coupled Coil Is in Close Proximity to the Primary Coil

We first examined the coupling behavior of two resonators tuned to the same frequency placed near each other. To mimic an inductively coupled coil–local primary coil configuration, a 5‐cm‐diameter coil and a 10‐cm‐diameter coil were arranged in a stacked configuration, with the 10‐cm coil serving as the primary coil. The inductively coupled coil was positioned 0.5 cm above a bottle phantom (11 cm diameter, 20 cm length, 1.4 g/L NaCl, 3.328 g/L NiCl_2_·6H_2_O solution), while the primary coil was placed approximately 1.5 cm above the inductively coupled coil. Both coils were tuned to 298 MHz (the Larmor frequency at 7 T), and the primary coil was matched to 50 Ω without the presence of the inductively coupled coil to represent a realistic operating condition.

**FIGURE 1:**
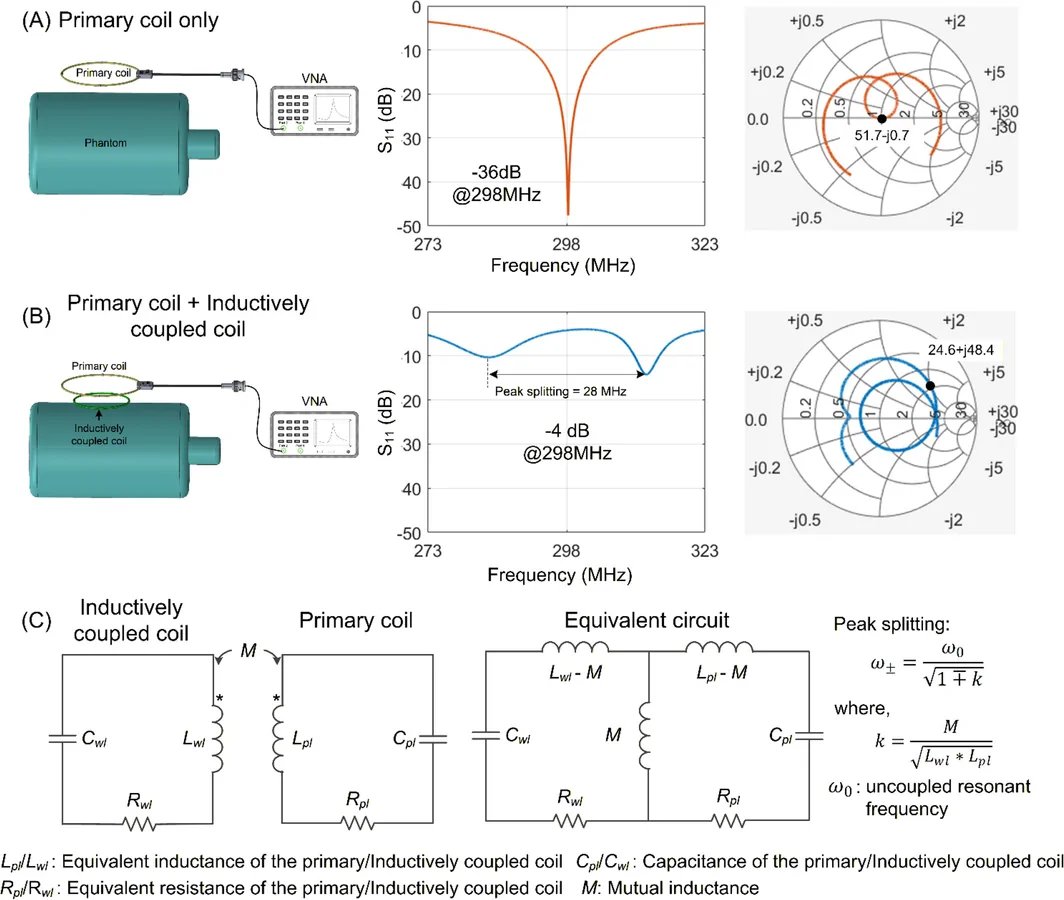
(A) Setup and results of measuring the impedance of a 10‐cm‐diameter circular 7 T RF coil on a bottle phantom. (B) Setup and results of the same coil (primary coil) when a smaller 5‐cm‐diameter inductively coupled coil was placed underneath the primary coil but above the phantom. The primary coil was not retuned or rematched after introducing the inductively coupled coil. (C) Simplified equivalent circuit model of the coupled inductively coupled and primary coils illustrating resonance splitting due to strong mutual coupling.

The input impedance of the primary coil was measured directly using one port of a well‐calibrated vector network analyzer (VNA, Keysight Technologies, USA). As shown in Figure 1A, the primary coil was well tuned and matched without the inductively coupled coil, exhibiting a return loss (S_11_) of −36 dB and an impedance of 51.7−j0.7 Ω. Upon introducing the inductively coupled coil, resonance splitting occurred (as evidenced by the split dips in the S_11_ plot), accompanied by significant impedance mismatch of the primary coil (24.6 + j48.4 Ω, only −4 dB return loss), as shown in Figure 1B. This behavior is well known in MRI coil engineering and reflects the strong mutual coupling between two closely spaced same‐frequency resonators [35].

This resonance splitting can be understood through an equivalent circuit model (Figure 1C), in which each coil is modeled as a series RLC resonator coupled through a mutual inductance *M*. When both coils are tuned to the same frequency *ω*_0_, the coupled system supports two eigenmodes with split resonances at $\omega_{\pm }=\omega_{0}/\sqrt{1\mp k}$ [36], consistent with the experimentally observed splitting in Figure 1B.

### 2.2 Theory of Coil Behavior

**FIGURE 2:**
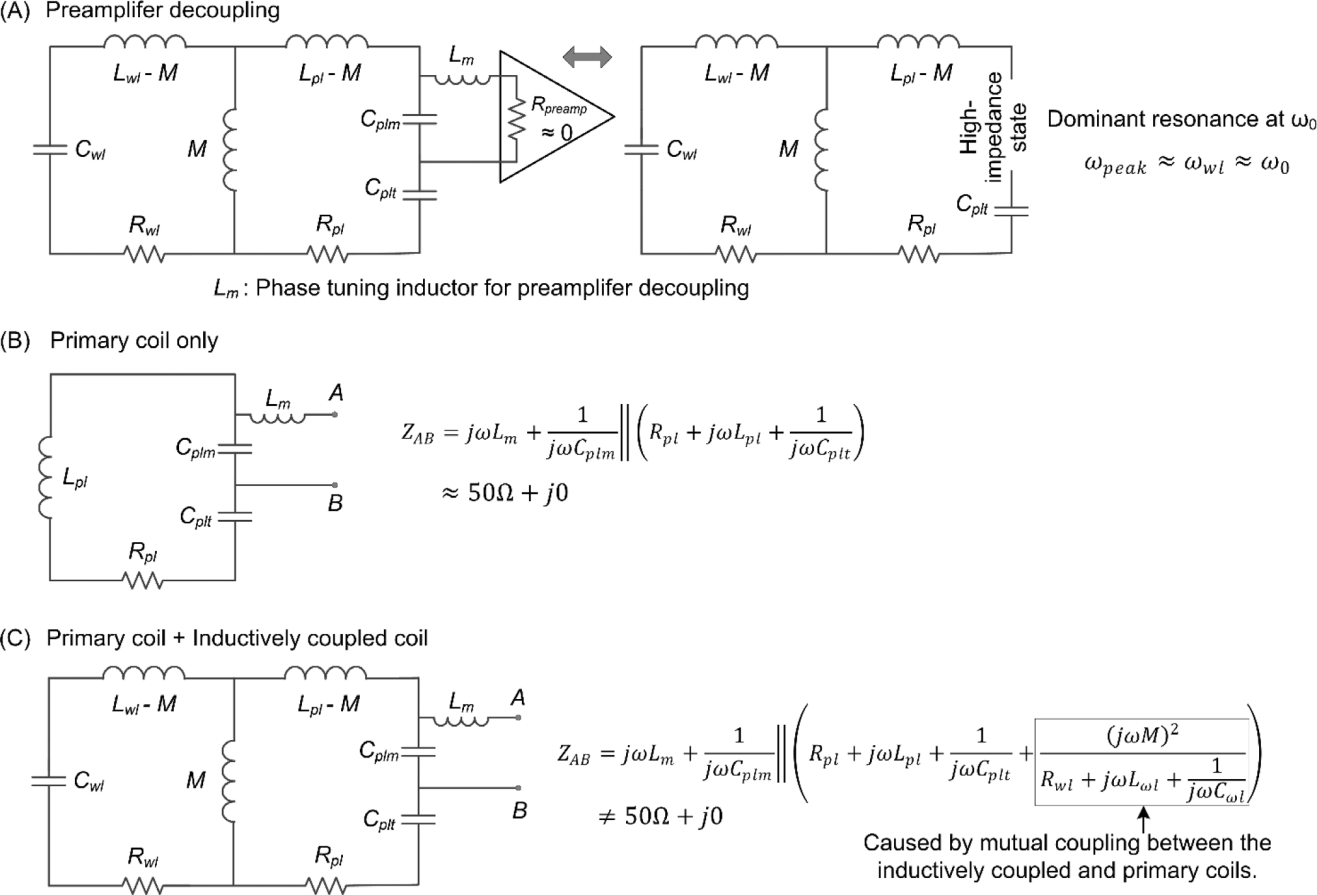
(A) Simplified equivalent circuit illustrating how preamplifier decoupling preserves the dominant resonance behavior of the inductively coupled coil. (B) Simplified circuit of the primary coil and the expression of impedance looking into the coil. (C) Simplified equivalent circuit of the primary coil with the inductively coupled coil, showing the additional reflected impedance term caused by mutual coupling between the inductively coupled and primary coils.

A simplified equivalent circuit illustrating this mechanism is shown in Figure 2A. Due to the low input impedance of the preamplifier connected to the primary coil (*R*_preamp_ ≈ 0 Ω), a high‐impedance condition is established at the coil terminals, formed by the parallel tank circuit of C_pl_ and L_m_ (Figure 2A). This effect, known as preamplifier decoupling [34], suppresses current flow in the primary coil, making it behave as an open circuit from the perspective of the inductively coupled resonator. It is analogous to preamplifier‐based decoupling used to minimize coupling between adjacent coil elements in a receiver array [34].

From the perspective of the primary coil, however, the inductively coupled resonator lacks preamplifier termination and still presents a resonant load. This interaction introduces an impedance mismatch at the primary‐coil port, as illustrated in Figure 2B,C. However, if the preamplifier performance, specifically the noise figure and gain, is robust to variations in source impedance, this mismatch results in only a minimal noise penalty.

### 2.3 Bench‐Test Validation

**FIGURE 3:**
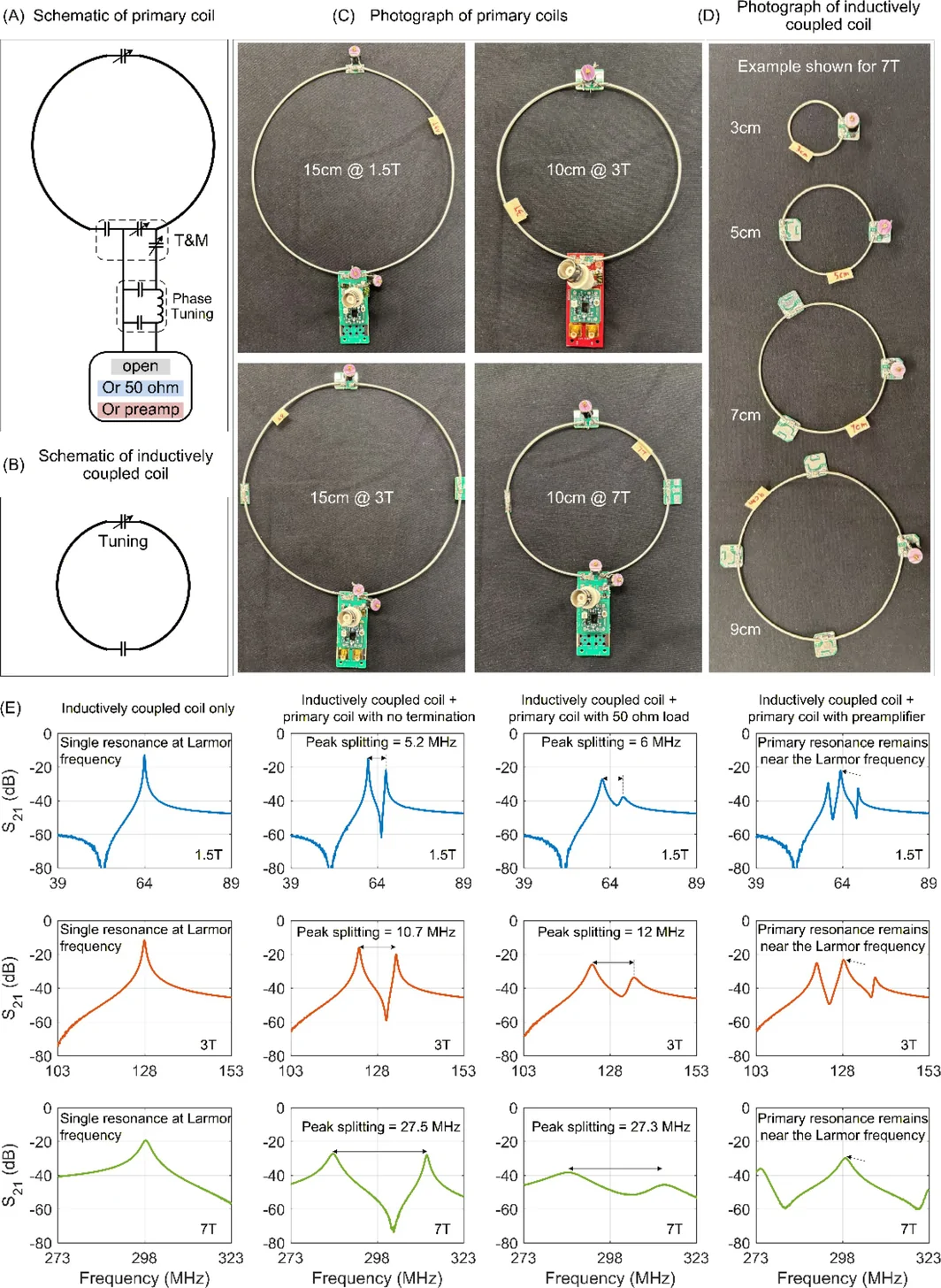
(A) and (B) Schematics of an example primary coil and an example inductively coupled coil. (C) Photographs of primary coils of different sizes for various static fields. (D) Photographs of inductively coupled coils of different sizes for 7 T. (E) Measured resonant peaks of inductively coupled coils when they are stacked with primary coils (under different termination conditions). Both coils were individually tuned to the Larmor frequency prior to stacking; no retuning was performed. The primary coil was matched to 50 Ω and achieved preamplifier decoupling via a π‐shaped network before the WanTcom preamplifier. Open‐circuit (no termination) and 50‐Ω terminations both cause severe resonance splitting and frequency shifts away from the Larmor frequency. However, the preamplifier termination effectively preserves the resonant peak at the Larmor frequency. Note that panel E shows the S_21_ of the secondary (inductively coupled) coil, monitored via well‐decoupled pick‐up probes under different primary‐coil termination conditions.

To comprehensively validate the proposed concept across multiple operating conditions, we fabricated circular primary coils of different sizes (diameters: 10 and 15 cm) and circular inductively coupled coils of different sizes (diameters: 3, 5, 7, and 9 cm) for operation at different static field strengths (1.5, 3, and 7 T), as shown in Figure 3A–D.

The inductively coupled coil is an L/C resonator constructed from 14‐AWG tin‐coated copper wire and a combination of fixed capacitors (Johanson Technology, EIA 1111) and trimmer capacitors (Sprague‐Goodman GYA36000). The primary coil has the same resonant structure but additionally incorporates a matching capacitor and a π‐shaped phase‐shifting network for preamplifier decoupling.

Bench tests included two measurements: (1) the resonant peaks of the inductively coupled resonator when positioned underneath the primary coil, measured under open‐circuit, 50‐Ω termination, and low‐input‐impedance preamplifier termination conditions; and (2) changes in the input impedance of the primary coil in the presence of an inductively coupled coil. The resonant behavior was evaluated using a pair of well‐decoupled pick‐up probes. During bench testing, the inductively coupled coil was positioned 0.5 cm above a body‐shaped phantom, while the primary coil was placed ∼1.5 cm above the inductively coupled coil. The phantom has an octagonal shape, with a height of ∼25 cm, a width of ∼35 cm, and a length of ∼40 cm, a conductivity of 0.6 S/m, and a relative permittivity of 78. MRI‐class preamplifiers from WanTcom (Chanhassen, MN, USA) were used in this study. Specifically, WMM1R5P, WMM3RP‐R5, WMM7RP were employed for operation at 1.5, 3, and 7 T, respectively. Note that the distance between the inductively coupled coil and the primary coil represents a typical configuration adopted in recent studies [23, 24, 25, 26, 27, 28] that employ a local receive coil rather than a body coil or a traveling‐wave antenna as the primary coil.

### 2.4 MRI Experimental Validation

MRI experiments were performed at 7 T using a 10‐cm‐diameter primary coil in combination with a 5‐cm‐diameter inductively coupled coil, using the configuration shown in Figure 1. The primary coil was tuned and impedance‐matched in the absence of the inductively coupled coil and was not retuned or rematched after introducing it. In addition to using the optimal cable length required to achieve effective preamplifier decoupling, we intentionally tested other cable lengths for which the preamplifier decoupling performance was degraded.

Both the primary and inductively coupled coils operated in receive‐only mode, incorporating either active or passive detuning circuits. The primary coil was connected to one port of a 16‐channel interface box with all remaining ports terminated with 50 Ω. RF transmission was provided by a birdcage volume coil (Nova Medical, USA), and its reference scale was kept constant across all scans. MRI experiments were conducted on a Philips 7 T whole‐body MRI scanner (Philips Healthcare, Best, the Netherlands).

Gradient‐echo (GRE) images were acquired using the following parameters to enable SNR evaluation under different experimental conditions: FOV = 150 × 150 mm^2^, nominal flip angle = 30°, TR/TE = 500/5 ms, in‐plane resolution = 1 × 1 mm^2^, slice thickness = 5 mm, number of averages = 1. Only the raw data from the active Rx channel were used for image reconstruction. Noise‐only scans, using the identical pulse sequence with the RF transmission disabled, were also performed for noise estimation.

## 3 Results

### 3.1 Bench Test Results

#### 3.1.1 Influence of the Primary Coil on Inductively Coupled Coil Resonance

Figure 3E illustrates the resonant peak variations of inductively coupled coils under different termination conditions. Without termination, the resonant peak clearly splits as expected for the strong mutual coupling. This split remains prominent even with a 50‐Ω termination (simulating a 50‐Ω preamplifier). Frequency shifts of up to 9.3% and resonant magnitude drops of ∼30 dB occur, which prevents the inductively coupled resonator from functioning as an effective local amplifier. With preamplifier termination, however, the resonant peak near the Larmor frequency remains stable, as shown in the black arrows. These results demonstrate that the low input impedance of the preamplifier effectively suppresses the influence of the primary coil and preserves the resonance behavior of the inductively coupled resonator. Note that preamplifier termination introduces two “extra peaks” in Figure 3. This occurs because of the narrowband nature of the preamplifier decoupling network. However, since MRI is a narrowband application, the central resonance remains sufficiently preserved for high‐quality imaging. Note that S_21_ (or S_12_) can also be measured by directly connecting the primary and secondary coils to two ports of the VNA (Figure S2). In this case, however, a normalized S_21_/S_12_ should be used rather than the raw measured values.

#### 3.1.2 Influence of the Inductively Coupled Coil on Primary Coil Impedance

MRI preamplifiers typically require a specific source impedance, usually 50 or 75 Ω, presented by the coil. For the specific preamplifier models used in this study, the target impedance is 50 Ω. In the receive system, the primary goal of impedance matching is to achieve optimum noise matching for the preamplifier rather than maximum power transfer.

**FIGURE 4:**
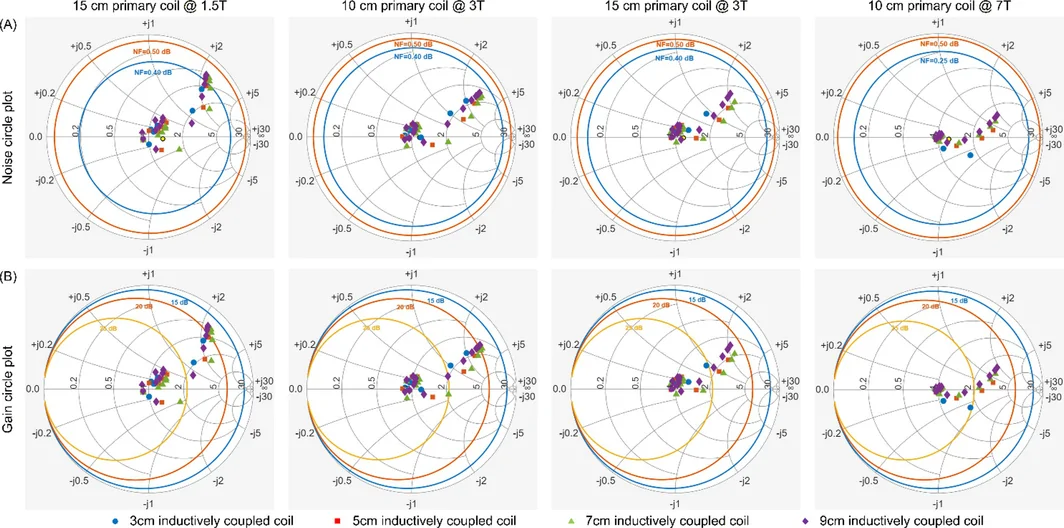
Measured impedances of the primary coil in the presence of the inductively coupled coil. The inductively coupled coil was moved to different relative positions compared to the primary coil. (A) Measured impedance with the noise figure circles overlaid. (B) Measured impedance with the gain circles overlaid. Across all tested resonator positions, the impedance excursions remain within the 0.2–0.5 dB noise‐figure contours, confirming that arbitrary resonator placement does not meaningfully degrade noise performance.

Figure 4 shows how the input impedance of the primary coils—initially matched to 50 Ω without an inductively coupled coil—shifts when an inductively coupled coil is introduced. For reference, manufacturer‐provided noise circles and gain circles are overlaid. At 3 and 7 T, where the noise circles are comparatively broad, the impedance excursions from all resonator placements remain within the 0.4 and 0.2 dB noise‐figure bounds, respectively, indicating negligible impact on noise performance. Even at 1.5 T, where the circle is tighter, the operating points still fall within the 0.5 dB contour. Overall, these results indicate that preamplifiers can tolerate substantial impedance variation without degrading SNR, supporting the use of an inductively coupled coil near the primary coil.

### 3.2 MRI Experimental Results

**FIGURE 5:**
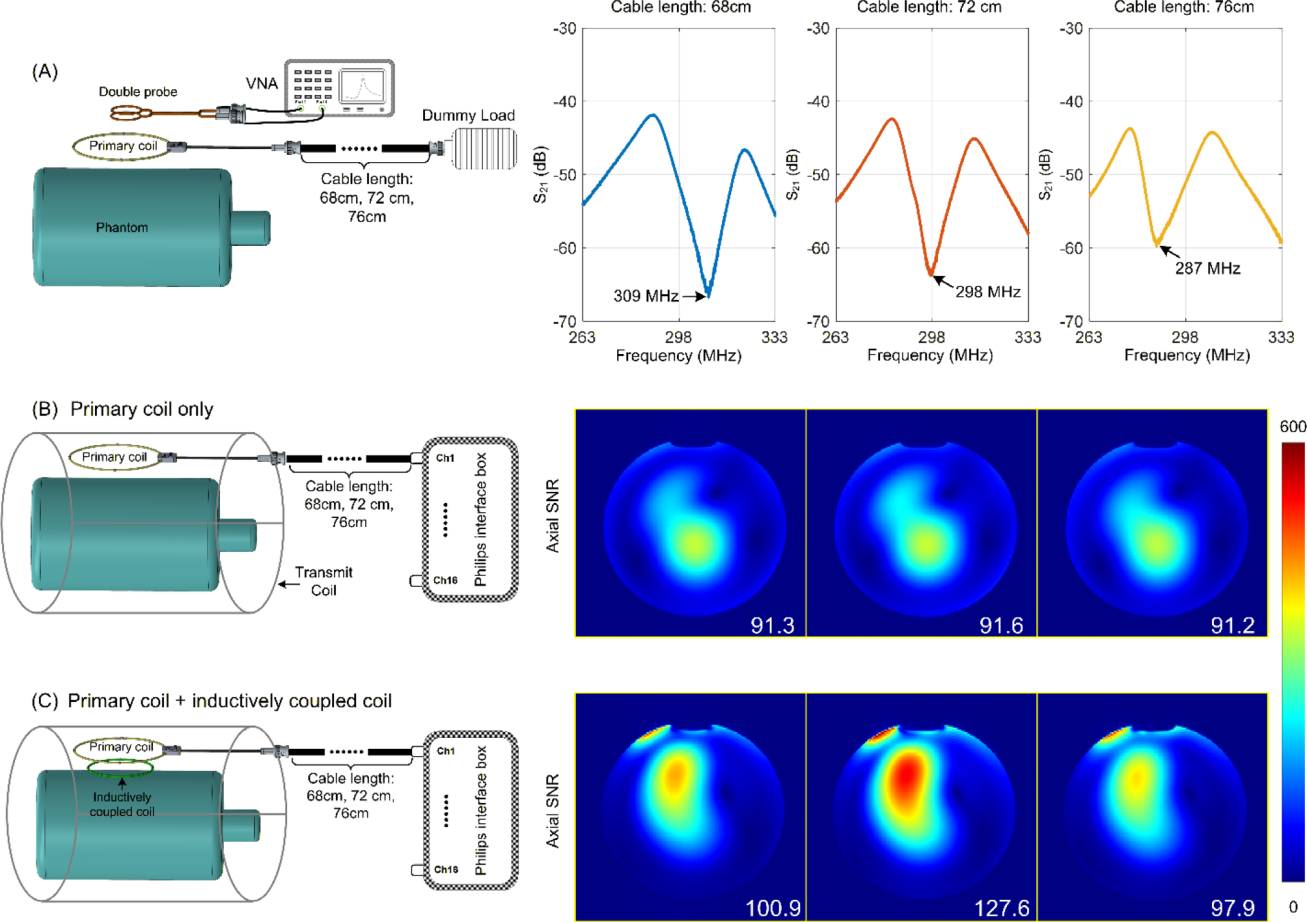
(A) Bench test results of a 7 T primary coil with different levels of preamplifier decoupling, achieved by using different cable lengths. (B) Axial SNR maps (based on GRE images) of the primary coil using different cable lengths. (C) Axial SNR maps of the primary coil using different cable lengths in the presence of a 5‐cm‐diameter inductively coupled coil. Average SNR values over the whole slice are marked in white in (B) and (C).

Figure 5A displays the S_21_ plots from the double probe (used to evaluate preamplifier decoupling) when the primary coil was connected to a dummy load via various cable lengths (68, 72, and 76 cm). The dummy load is a predominantly reactive load that mimics the preamplifier input impedance in its active state. The 72 cm cable corresponds to the primary coil being tuned for optimal preamplifier decoupling (with the S_21_ dip precisely at 298 MHz). In contrast, the 68 and 76 cm cables were used to intentionally misalign the preamplifier decoupling; the S_21_ dip shifted higher to 309 MHz and lower to 287 MHz, respectively.

Figure 5B shows the measured SNR of the primary coil without the inductively coupled resonator. In this case, varying the preamplifier decoupling (via cable length) does not cause any SNR changes (< 1% change). This result is expected, as cable lengths do not alter the coil's 50 Ω impedance. Figure 5C shows the measured SNR of the primary coil with the inductively coupled resonator. In this case, the 68 and 76‐cm‐long cables showed noticeable SNR drops of 21% and 23%, respectively, compared to the 72‐cm‐long baseline. These results are consistent with bench testing and confirm that preamplifier decoupling in the primary coil is critical for inductively coupled coil applications, particularly when the two are strongly coupled. Notably, Figure 5B,C were acquired under identical conditions, and the SNR enhancement from the inductively coupled resonator is clearly observed. Given that the primary coil is only ∼1.5 cm from the secondary coil, this gain is largely attributable to the smaller size and improved local sensitivity of the inductively coupled coil.

## 4 Discussion and Conclusion

In this work, we demonstrate that preamplifiers on the primary coil mitigate over‐coupling effects in the presence of inductively coupled secondary coils. Traditionally, strong coupling has been associated with resonant peak splitting—particularly in the secondary coil—and increased noise due to impedance mismatch in the primary coil. Our results show that, with appropriate preamplifier decoupling, these effects are largely suppressed. This enables stable operation in strong‐coupling regimes, even at close proximity, without performance penalties, thereby simplifying design and improving practical applicability.

Although low‐impedance preamplifiers are widely used and their decoupling effect is well known to RF engineers who design array coils, their specific role in preserving the resonant behavior of nearby inductively coupled coils has not been explicitly described. In addition, their robustness to coil impedance variation has not been systematically studied. While one might expect impedance deviations to degrade noise performance, our results indicate that this is not the case under the conditions examined.

This study highlights the critical role of the preamplifier in the over‐coupling regime. At larger separations (e.g., > 12 cm for a 10‐cm primary coil and a 5‐cm secondary coil at 7 T; see Figure S1), the system transitions to the under‐ or critical‐coupling regime, where the influence of the preamplifier becomes less significant. This work focuses on preamplifiers from WanTcom. Low input impedance is a general feature of modern MRI preamplifiers, and the findings are expected to be broadly applicable.

50‐Ω preamplifiers are still used in some preclinical scanners and ultra‐low‐field MRI systems. In such cases, strong coupling can be managed by retuning the primary coil and/or the secondary coil so that the desired resonant peak aligns with the Larmor frequency, as described by Wright et al. and Hoult & Tomanek [19, 20]. However, retuning is inherently geometry‐dependent, as the optimal frequency offset varies with mutual inductance and therefore with coil separation and relative positioning. For ultra‐low‐field MRI systems, however, the implications of repositionable inductively coupled coils operating with 50‐Ω preamplifier inputs remain an area for future investigation.

When the primary coil is terminated with a low‐input‐impedance preamplifier, coupling becomes effectively asymmetric: the inductively coupled coil carries real current and transfers flux to the primary coil, while current induced in the primary coil is minimized. Combined with bench measurements showing preservation of the inductively coupled coil resonance, this indicates that closer spacing between the primary coil and the inductively coupled coil improves SNR. As shown in Figure S3, reducing the separation between the two coils consistently increases SNR. At 7 T, this is further enabled by the large noise‐figure circles of the preamplifier, which make the system insensitive to impedance mismatch at the primary coil. It is also worth noting that the distance between the inductively coupled coil and the sample directly affects SNR through the filling factor: closer is better. When a tradeoff exists between proximity to the primary coil and proximity to the sample, prioritizing the latter is generally the better choice.

This work resolves a long‐standing question of why, in practice, over‐coupling between an inductively coupled coil and a primary coil does not lead to performance degradation by showing that modern low‐input‐impedance preamplifiers fundamentally reshape coupled‐coil dynamics and relax traditional coupling constraints. Looking forward, these findings support the broader adoption of inductively coupled coils in emerging applications, such as wearable and anatomy‐conforming coils, where flexible, cable‐free RF solutions are increasingly valuable.

## Funding

This work was supported by the National Institutes of Health (R01 EB031078, R21 EB029639, R21 EB037763, S10 OD030389).

## Supporting information


**Figure S1:** Demonstration of different coupling regimes between an inductively coupled coil and a primary coil. The inductively coupled coil and primary coil had diameters of 5 cm and 10 cm, respectively, and were both tuned to the Larmor frequency at 7 T (298 MHz). From a theoretical perspective, for two resonators tuned to the same frequency, the coupling regime (under, critical, and over‐coupling) is determined by comparing the coupling coefficient (*k*) to the inverse of the quality factor (1/Q). Specifically, the system is under‐coupled when k<1/Q, critically coupled when k=1/Q, and over‐coupled when k>1/Q. Form a practical perspective, the coupling regimes are identified based on the resonance profile: a single, well‐defined peak (under‐coupling); a flattened or broadened peak indicating the onset of splitting (critical coupling); and a clearly split peak (over‐coupling). These conditions can be readily measured using a pair of well‐decoupled probes. As shown in this figure, the inductively coupled coil is under‐coupled when the separation from the primary coil is greater than ∼12 cm, critically coupled at approximately 12 cm, and over‐coupled at separations below 12 cm.
**Figure S2:** Measured S‐parameter versus frequency plots of a primary coil and an inductively coupled coil as their relative position changes. When the impedance of both coils remains close to 50 Ω (S_11_ < −10 dB), S_21_ increases with stronger coupling, as expected. However, under strong coupling conditions where clear peak splitting is observed, the simple S_21_ value becomes an unreliable indicator of coupling strength. In such cases, a normalized metric should be used for more accurate characterization.
**Figure S3:** Effect of coil separation on SNR. (A) Axial SNR maps of a cube phantom acquired with a 5‐cm‐diameter inductively coupled coil at separation distances ranging from 7 to 42 mm in 7‐mm increments. (B) Schematic of the experimental setup. The inductively coupled coil and phantom were fixed, and the separation distance *d* was adjusted by moving the primary coil. (C) Mean ROI SNR near the inductively coupled coil as a function of separation distance. The mean SNR increased as the separation distance decreased, indicating that closer spacing improves SNR under preamplifier decoupling.

## Data Availability

The data that support the findings of this study are available from the corresponding author upon reasonable request.
