# Supplementary material for: Revisiting Inductively Coupled Wireless Coils in MRI: Mitigating Over‐Coupling With Preamplifiers
Source: Magn Reson Med. 2026 Jun 21;96(5):2553–61. doi: 10.1002/mrm.70485 (PMC13527287; doi:10.1002/mrm.70485)
Supplement: Supplementary file 1 [file MRM-96-2553-s001.docx]

**Supplementary Materials**

The Supplementary Information includes 3 Supplementary Figures.

**Revisiting Inductively Coupled Wireless Coils in MRI: Mitigating Over-Coupling with Preamplifiers**

*Ming Lu ^a*^, John C. Gore ^a,b,c^, Xinqiang Yan ^a,b,d†^*

a. Vanderbilt University Institute of Imaging Science, Vanderbilt University Medical Center, Nashville, TN, 37232, USA
b. Department of Radiology and Radiological Sciences, Vanderbilt University Medical Center, Nashville, TN, 37232, USA
c. Department of Biomedical Engineering, Vanderbilt University, Nashville, TN, 37232, USA
d. Department of Electrical and Computer Engineering, Vanderbilt University, Nashville, TN, 37232, USA

**Corresponding Author:**

Xinqiang Yan, Ph.D.

Vanderbilt University Institute of Imaging Science

1161 21^st^ Avenue South
Medical Center North, D-2205
Nashville, TN 37232-2310

Email: xinqiang.yan@vumc.org

**Figure S1**	Demonstration of different coupling regimes between an inductively coupled coil and a primary coil. The inductively coupled coil and primary coil had diameters of 5 cm and 10 cm, respectively, and were both tuned to the Larmor frequency at 7 T (298 MHz). From a theoretical perspective, for two resonators tuned to the same frequency, the coupling regime (under‑, critical, and over‑coupling) is determined by comparing the coupling coefficient (*k*) to the inverse of the quality factor (1/Q). Specifically, the system is under‑coupled when $k<1/Q$, critically coupled when $k=1/Q$, and over‑coupled when $k>1/Q$. Form a practical perspective, the coupling regimes are identified based on the resonance profile: a single, well-defined peak (under‑coupling); a flattened or broadened peak indicating the onset of splitting (critical coupling); and a clearly split peak (over‑coupling). These conditions can be readily measured using a pair of well‑decoupled probes. As shown in this figure, the inductively coupled coil is under‑coupled when the separation from the primary coil is greater than ~12 cm, critically coupled at approximately 12 cm, and over‑coupled at separations below 12 cm.

**Figure S2**	Measured S‑parameter versus frequency plots of a primary coil and an inductively coupled coil as their relative position changes. When the impedance of both coils remains close to 50 Ω (S₁₁ < −10 dB), S₂₁ increases with stronger coupling, as expected. However, under strong coupling conditions where clear peak splitting is observed, the simple S₂₁ value becomes an unreliable indicator of coupling strength. In such cases, a normalized metric should be used for more accurate characterization.

**Figure S3**	Effect of coil separation on SNR. (A) Axial SNR maps of a cube phantom acquired with a 5-cm-diameter inductively coupled coil at separation distances ranging from 7 to 42 mm in 7-mm increments. (B) Schematic of the experimental setup. The inductively coupled coil and phantom were fixed, and the separation distance d was adjusted by moving the primary coil. (C) Mean ROI SNR near the inductively coupled coil as a function of separation distance. The mean SNR increased as the separation distance decreased, indicating that closer spacing improves SNR under preamplifier decoupling.
